# Probability of myopia in children with high refined carbohydrates consumption in France

**DOI:** 10.1186/s12886-020-01602-x

**Published:** 2020-08-18

**Authors:** Claire Berticat, Sonia Mamouni, Angelique Ciais, Max Villain, Michel Raymond, Vincent Daien

**Affiliations:** 1 ISEM, University of Montpellier, CNRS, EPHE, IRD, Montpellier cedex 05, France; 2 Department of Ophthalmology, Gui De Chauliac Hospital, Montpellier, France; 3 Department of Orthoptist, Gui De Chauliac Hospital, Montpellier, France; 4 Neuropsychiatry: Epidemiological & Clinical Research, University of Montpellier, INSERM, Montpellier, France

**Keywords:** Myopia, Risk factors, Diet, Refined carbohydrates, Sugar, Starch, Screen time, Outdoor time, Children

## Abstract

**Background:**

Evaluate risk factors for paediatric myopia in a contemporary French cohort taking into account consumption of refined carbohydrates (starches and sugars).

**Methods:**

An epidemiological cross-sectional study was conducted between May 2017 and May 2018.

Two hundred sixty-four children aged 4 to 18 years attending the Centre Hospitalier Universitaire Gui de Chauliac in Montpellier were recruited. Ophthalmologic or optometric cycloplegic refraction were measured. Evaluated risk factors for myopia were collected, including family history of myopia, outdoor time, reading time, screen time, physical activity, and consumption of refined carbohydrates. Association between the probability of at least one eye showing myopia (defined as < 0 D) and frequency of refined carbohydrates consumption adjusted for risk factors and control factors was tested.

**Results:**

Overall, 86/264 (32.6%) children investigated showed myopia in at least one eye. We included 180 children exhibiting refraction < 3 D in both eyes: 88 (48.9%) girls and 92 (51.1%) boys. The consumption of refined carbohydrates significantly increased the probability of myopia for girls (odds ratio [OR] = 1.07; 95% confidence interval [CI], 1.02–1.13; *P* = 0.009) but decreased it for boys (OR = 0.94; 95% CI, 0.89–0.98; *P* = 0.011). The probability of myopia was marginally increased with increased screen time (OR = 2.32; 95% CI, 0.94–6.47; *P* = 0.083). Outdoor time seemed marginally protective (OR = 0.74; 95% CI, 0.54–1.01; *P* = 0.057).

**Conclusion:**

Refined carbohydrates consumption could be associated with child myopia, with increased probability for girls and unexpected reduced probability for boys, possibly due to the fact that frequency of carbohydrates consumption do not really capture boy’s chronic hyperglycemia, boys being more physically active than girls at all ages. Some known risk/protective factors of myopia were marginally significant: screen time (risk) and outdoor time (protective). This study reinforces the belief that modifiable risk factors for myopia could be targets for future public health actions.

## Background

Myopia is a multifactorial refractive disorder characterised by blurry distance vision with eyes displaying steeper corneal curvature and/or longer axial length as compared with emmetropes [1]. High myopia (usually defined as < − 6 D) is a risk factor for potentially blinding complications such as retinal detachment, subretinal neovascularisation, early cataract and glaucoma [2].

Myopia has become a significant public health problem, with a substantial increase in prevalence worldwide [3]. For example, in China, the proportion of people with myopia increased from 20% in the 1970s to 90% in 2018 [4]. In 2010, 28% of the world’s population was myopic and a group of world health experts projected that with the current trends, half of the world’s population will be affected by myopia in 2050 [5].

With the fast time-scale increase in myopia (less than 2 to 3 generations), non-genetic associated factors are being identified [6, 7]. Time spent doing close eye work (near-work), duration of study time and level of education are most frequently cited as the main environmental factors underlying the development of myopia [8–10]. Outdoor time (exposure to natural light) but not physical activity is described as a protective factor because children spending more time outside show less incidence of myopia [11–13]. To control for genetic variability, Ramessur et al. (2015) compared refractions in several pairs of homozygous twins and showed that the most myopic twin was the one who spent the least time outside [14].

Other possible factors were previously proposed, but were later discarded. For example, in 1956, Gardiner suggested dietary involvement in the pathophysiology of myopia: a comparison of the diet of 33 active myopic and 251 stable myopic individuals showed increased consumption of lipids and carbohydrates in the active group [15]. Almost 2 decades ago, Cordain et al. (2002) first proposed that via hyperinsulinism, consumption of refined carbohydrates (starches and sugars) could be involved in the development of juvenile-onset myopia: the interaction between hyperinsulinism and hormonal regulation of eye growth could increase the elongation of the axial eyeball [16]. This hypothesis has been supported by more recent evidence [17–23]. Of note, the dietary hypothesis and the near-work hypothesis cannot be tested independently, because the type and quantity of diet is associated with physical activities, which may be negatively correlated with time spent on near-work [24–26]. To our knowledge, only one study considered both effects jointly by controlling also for outdoor activities with the conclusion that axial length and sugary diet were not associated [27]. However, in this study, sugars and starches consumption were considered separately and without distinction of their refined origin, possibly reducing the ability to detect an effect. Moreover, this study found a statistically significant link between consumption of saturated fatty acids as well as cholesterol level and increase in axial length. Saturated fat is a known antagonist of insulin and a contributor to insulin resistance [28], so these findings lend some support to the hyperinsulinemic theory of Cordain et al. [16]. Thus, the contribution of these two hypotheses, near-work or diet, to the emergence of juvenile onset myopia is unclear.

The aim of this study was to simultaneously test modifiable risk factors suspected to be involved in the development of myopia in children, taking into account nutritional factors especially the consumption of refined carbohydrates.

## Methods

### Design

All children age 4 to 18 years who attended the University Hospital Center of Montpellier from May 2017 to May 2018 were considered. We excluded children with organic ophthalmological pathologies such as cataract, glaucoma, retinoblastoma, and Coat’s disease but included those with a history of amblyopia and functional strabismus.

### Measurements

All included children underwent a complete ophthalmologic examination, including refraction under cycloplegia, slit-lamp examination and dilated fundus examination. Children with refraction error ≥ 3 D in at least one eye were excluded because those individuals were considered as moderate to high hyperopic and thus could not be used as control (i.e. non myopic). The resulting children were considered myopic (< 0 D for one or both eyes; using a cut-off of - 0.5 D does not change qualitatively the results) or controls (non-myopia for both eyes). Parents completed the study questionnaire to collect the following information on the child: sex (M, F), age (year), height (cm), weight (kg), whether the mother or father was myopic (yes/no), reading time (hours per day), screen time (tablets/cell phones, video games, computers etc.; hours per day), outdoor time (hours per day), physical activities (yes/no) and refined carbohydrates consumption by using a food frequency questionnaire (see Additional files 1 and 2) .

#### Refined carbohydrates consumption

Refined carbohydrates intake was measured by summing the frequency of weekly consumption of high glycaemic load products reported in the food frequency questionnaire. Reported frequencies were transformed in weekly frequencies as follows: 0 for never, 0.5 for less than once a week, 1 for once a week, 2.5 for two to three times a week, 5 for four to six times a week, 7 for every day. This food frequency questionnaire was adapted from the one used in the French national cohort Constances, designed to reflect intake in the French population, selected food items being compliant with the nutritional guidelines from the French National Nutrition and Health Program (PNNS) [29]*.*

#### Cycloplegic refraction

Cycloplegia was obtained with administration of cyclopentolate (Skiacol, Alcon, Fort Worth, TX, USA) or IsoptoAtropine (Alcon, Forth Worth, TX, USA) at 0.5% for children age 4 to 12 years and 1% for children age 12 to 18 years as recommended by French health authorities. Instillation protocols were those validated in current practice: 1 h, 55 and 50 min before measurement for Skiacol and twice a day for 5 days before measurement for IsoptoAtropine. Refraction was measured by using a NIDEK TONOREF II Auto Refractometer (Nidek medical, Settimo Milanese, Italy) in children age 12 to 18 years and the Retinomax 2 Portable Self-Refractometer (Visionix, Bensenville, IL, USA) when the child’s cooperation did not allow use of the TONOREF II.

### Statistical analyses

All statistical analyses involved using R v3.5.2 (www.r-project.org) with MASS v.7.3–51.1 [30]. Logistic regression was used to analyse the probability of being myopic, estimating odds ratios (ORs) and 95% confidence intervals (CIs). The binary response variable corresponded to spherical refractive error < 0 D versus ≥0 D for at least one eye. Explanatory variables were reading time per day (quantitative), screen time per day (quantitative), time spent outside per day (quantitative) and refined carbohydrates consumption per week (quantitative). Control variables were z-scores for body mass index (BMI [weight/height^2^], based on the growth reference for age 5 to 19 years from the World Health Organization https://www.who.int/growthref; quantitative), mother and father myopia (categorical), sex (categorical), age (quantitative) and sport (categorical). All quantitative variables were centered. The following interactions were analysed beforehand: age with sex, screen time, reading time, outside time, and sport; sex with screen time, reading time, outside time, and sport; and refined carbohydrates consumption with sport, sex, outside time, and age. The significance of each term was assessed from the model including all the other variables by using a likelihood ratio chi-square test. *P* < 0.10 was considered statistically significant for interactions. The variance inflation factor was calculated by the function vif in the R package car [31].

## Results

### Population description

Among 264 children with age 4 to 18 years, 86 (32.6%) were myopic in at least one eye, with an unequal distribution by sex (girls: 49/128 [38.3%], boys: 38/136 [27.9%]). We included 180 children exhibiting refraction < 3 D in both eyes in the study: 88 (48.9%) girls and 92 (51.1%) boys. The mean age of children was 9.5 years old. The description of their characteristics is in Table 1 and the age distribution is in Table 2. The description of vision status is in Table 3. Two categories of vision status were considered: myopic in one or both eyes (*N* = 86; 49 girls, 37 boys; Table 3) and non-myopic in both eyes (*N* = 94; 39 girls, 55 boys).

**Table 1:** Characteristics of children included in the study (*n* = 180)

	Girls (*N* = 88)						Boys (*N* = 92)					
	Myopic^a^ (*N* = 49)			Non-myopic^b^ (*N* = 39)			Myopic^a^ (*N* = 37)			Non-myopic^b^ (*N* = 55)		
	Mean	SD	Range	Mean	SD	Range	Mean	SD	Range	Mean	SD	Range
Age (years)	10.43	4.06	4–17	8.31	3.32	4–17	10.89	3.72	4–17	8.31	3.65	4–18
BMI^c^ Z-score	0.21	1.47	− 3.60–3.57	0.22	0.92	−1.27–2.03	−0.02	1.50	−5.03–2.45	−0.03	1.31	−3.38–2.45
Sphere right eye (D)	−2.62	2.57	−8.50–1.50	0.94	0.89	0.00–2.75	−3.37	2.78	−12.00–0.00	1.01	0.92	0.00–2.75
Sphere left eye (D)	−2.62	2.91	−10.25–2.25	0.88	0.86	0.00–2.5	−3.21	2.77	−9.75–0.00	1.00	0.99	0.00–2.75
Outdoor time (hr/day)	2.48	1.40	0.57–6.43	2.63	1.38	1.28–7.14	2.31	1.22	0.57–5.86	2.89	1.65	1.00–7.14
Reading (hr/day)	0.73	0.21	0.50–1.00	0.68	0.21	0.50–1.00	0.71	0.20	0.50–1.00	0.65	0.20	0.50–1.00
Screens (hr/day)	2.63	0.61	1.00–4.00	2.29	0.48	1.00–3.25	2.78	0.57	2.00–4.00	2.47	0.53	0.50–3.50
Refined carbohydrates consumption (frequency/week)	41.94	13.56	13.00–86.00	35.04	11.06	10.00–53.50	35.03	12.04	0.50–56.00	40.78	10.98	11.50–56.50

^a^Myopic on one or both eyes (D < 0)

^b^Non myopic on both eyes (0 ≤ D < 3)

^c^*BMI* Body mass index

**Table 2:** Distribution of the children by age class

Age, years	Girls (*N* = 88)	Boys (*N* = 92)	All (*N* = 180)
4–6	26 (29%)	31 (34%)	57 (32%)
7–10	28 (32%)	28 (30%)	56 (31%)
11–18	34 (39%)	33 (36%)	67 (37%)
All	88 (100%)	92 (100%)	180 (100%)

**Table 3:** Vision status of children

Vision status for both eyes	Girls (*N* = 88)	Boys (*N* = 92)	All (*N* = 180)
Myopic^a^ both eyes	41 (46%)	35 (38%)	76 (42%)
Myopic and emmetropic^b^	3 (3%)	2 (2%)	5 (3%)
Myopic and hypermetropic^c^	6 (7%)	0 (0%)	5 (3%)
Emmetropic and hypermetropic	1 (1%)	2 (2%)	3 (2%)
Emmetropic both eyes	13 (15%)	18 (20%)	31 (17%)
Hypermetropic both eyes	25 (28%)	35 (38%)	60 (33%)
All (myopic one or both eyes)	49 (56%)	37 (40%)	86 (48%)
All (non-myopic in both eyes)	39 (44%)	55 (60%)	94 (52%)
All	88	92	180

^a^refraction error < 0D

^b^refraction error = 0D

^c^0D < refraction error < 3D

### Effects on probability of myopia

**Fig. 1:**
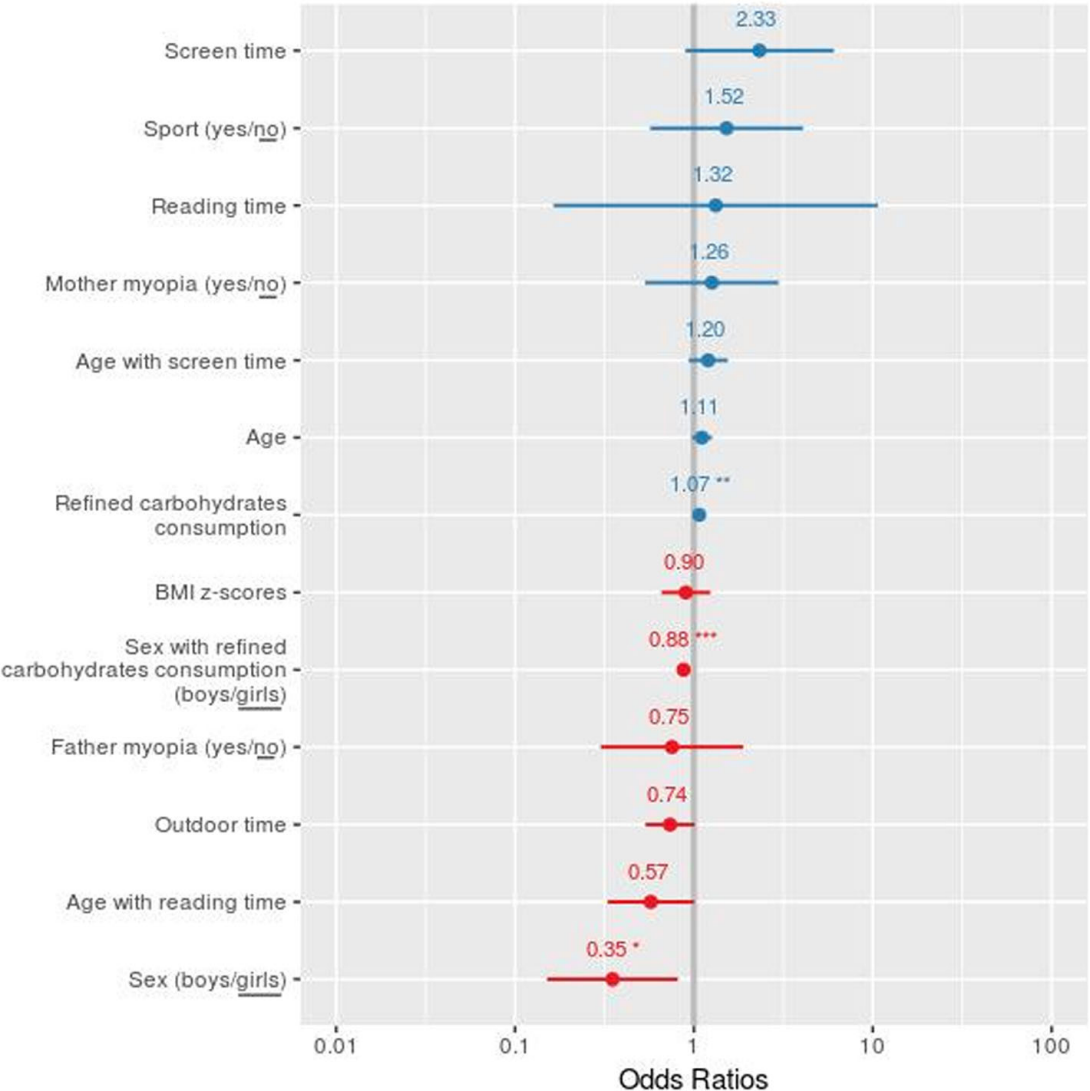
Adjusted odd ratios and 95% confidence intervals for the model studying the impact of risk and control variables on the probability of myopia in children. For categorical data, the estimates are for one category compared to the reference category (underlined term). * *P* < 0.05 ** *P* < 0.01 *** *P* < 0.001

Only the interactions age with screen time, age with reading time and sex with refined carbohydrates consumption were significant (χ^2^ = 3.74 df = 1 *P =* 0.053, χ^2^ = 5.50 df = 1 *P =* 0.019 and χ^2^ = 12.7 df = 1 *P =* 0.0003, respectively) and were thus kept in the final model. The final model (Table 4, Fig. 1) explained 22% of the total deviance and the variance inflation factor was < 2.5, indicating weak multicollinearity between covariables, and did not need to be accounted for [31]. The effect of refined carbohydrates consumption on myopia differed by sex (β = − 0.133; *P* < 0.001; OR = 0.87; 95% CI, 0.81–0.94, Table 4, Fig. 1). The consumption of refined carbohydrates significantly increased the probability of myopia for girls (*β* = 0.068; *P* = 0.009; OR = 1.07; 95% CI, 1.02–1.13) and decreased it for boys (*β* = − 0.065; OR = 0.94; 95% CI, 0.89–0.98; *P* = 0.011). Myopia was associated but not significantly with screen time (*β* = 0.844; OR = 2.32; 95% CI, 0.89–6.05; *P* = 0.083), and outdoor time seemed protective but was not significant (*β* = − 0.307; OR = 0.74; 95% CI, 0.54–1.01; *P* = 0.057). The age with reading time interaction was marginally significant (*β* = − 0.555; OR = 0.57; 95% CI, 0.33–1.00; *P* = 0.050), which indicates less myopia with increased age and reading time. Male sex was inversely associated with myopia (*β* = − 1.047; OR = 0.35; 95% CI, 0.15–0.8; *P* = 0.015) all things being equal. Parental myopia had no significant influence, either when myopia of each parent were considered separately (for both, *P* > 0.50), or when the number of myopic parent (0, 1 or 2) was considered as a quantitative variable (*P* = 0.973).

**Table 4:** Association of risk variables on the probability of myopia in children. For categorical data, the estimates are for one category compared to the reference category (underlined term). For each variable, the estimate β, standard error of the mean (SE), Z value and corresponding *P*-value, Odd-ratio with 95% confidence interval are given. Bold characters indicates significant (*P* < 0.05) effects. Italic characters indicates trends (*P* < 0.1)

	*β*	SE	z value	*P*(>|z|*)*	OR (95% CI)
Intercept	−0.253	0.548	0.055	0.956	
Refined carbohydrates consumption (frequency/week)	0.068	0.026	2.615	**0.009**	1.071 (1.017–1.127)
Screen time (hr/day)	0.844	0.488	1.731	*0.083*	*2.326* (0.894–6.049)
Reading time (hr/day)	0.281	1.065	0.264	0.792	1.325 (0.164–10.694)
Outdoor time (hr/day)	−0.307	0.161	−1.903	*0.057*	0.736 (0.536–1.009)
Mother myopia (yes/no)	0.231	0.438	0.528	0.598	1.260 (0.534–2.975)
Father myopia (yes/no)	−0.282	0.467	−0.604	0.546	0.754 (0.301–1.885)
Sport (yes/no)	0.421	0.501	0.839	0.401	1.523 (0.570–4.073)
Age (years)	0.103	0.067	1.548	0.122	1.109 (0.973–1.263)
Sex (boys/girls)	−1.047	0.430	−2.438	**0.015**	0.351 (0.151–0.814)
BMI z-scores	−0.103	0.160	− 0.645	0.519	0.902 (0.658–1.235)
**Interactions**					
Age with screen time	0.183	0.129	1.424	0.154	1.201 (0.933–1.547)
Age with reading time	−0.555	0.283	−1.959	*0.050*	0.574 (0.330–1.000)
Sex with refined carbohydrates consumption (boys with refined carbohydrates consumption /girls with refined carbohydrates consumption)	−0.133	0.037	−3.565	**3 10**^**−4**^	0.875 (0.814–0.942)

## Discussion

This study aimed at evaluating conjoint modifiable risk factors involved in the development of myopia in a French paediatric population, including the impact of consumption of refined carbohydrates. We found an association between child myopia and this type of diet. Risk of myopia was increased for girls with refined carbohydrate consumption but decreased for boys. Some already known risk/protective factors of myopia were concurrently detected: screen time was marginally associated with increased probability of myopia and outdoor time seemed protective.

### Carbohydrates consumption and myopia

Since the seminal study of Cordain (2002), very few studies had focused on the possible effect of refined carbohydrate consumption on myopia [17, 27]. Here we found a positive association between refined carbohydrates consumption and prevalence of myopia in girls aged 4 to 18 years but a negative one in boys. Refined carbohydrates (refined starches and sugars) are rapidly absorbed into the bloodstream, inducing a high peak of insulin (hyperinsulinemia), The more a carbohydrate is refined, the larger is the glycaemic and insulinaemic responses which can be measured by the glycemic load [32]. Fructose is an exception, being metabolised independently of insulin action in the liver. However, chronic hyperinsulinemia and fructose metabolism leads to insulin resistance [16, 33–35] and compensatory hyperinsulinemia [36–38], associated with many health challenging condition [39, 40]. Cordain et al. (2002) and recently Galvis et al. (2016) suggested that this hyperinsulinism could increase the elongation of the globe via the promotion of increased insulin-like growth factor-1 (IGF-1) and decreased insulin-like growth factor binding protein-3 (IGFBP-3) action in scleral fibroblasts [16, 17].

The increase in prevalence of myopia observed in all countries or populations that have adopted a sugar-rich western diet supports this hypothesis [3, 41, 42], even if several social and/or genetics factors are modulating this correlation. For example, Alaskan Inuit moved from a 0–2% prevalence of myopia to > 50% prevalence in a single generation as a result of a westernised lifestyle including eating habits [41]. Morgan and Munro (1973) reported similar patterns in several ethnic groups of the Yukon and Northwest regions of Canada, where myopia prevalence rates were also age-dependent [42]. Wong et al. (1993) found an increase in myopia prevalence (18.4%) among urban Hong Kong fishermen who had not attended school, which suggests that lifestyle factors such as changes in eating habits could be involved in the prevalence of myopia [43].

Unexpectedly, we showed a negative association between refined carbohydrate consumption and myopia for boys. The result that carbohydrates play different roles in boys and girls was unexpected, and not previously reported. This result cannot be attributed to quantitative difference in consumption between the sexes, as the sex had no significant influence on refined carbohydrate consumption recorded (*P* = 0.63, details not shown), although the qualitative difference of high refined carbohydrates consumption was not considered here. This finding could be the due to the frequency of carbohydrates consumption not really capturing boy’s chronic hyperglycemia because boys are more physically active than girls at all ages [44].

### Outdoor time and myopia

Time spent outside seemed a protective factor in myopia, in agreement with several studies [12, 13, 45], although the association was here marginally significant. It has been shown in children that the elongation of the globe, and the subsequent increase in myopia, was greater in winter than summer [46]. This effect could result from the increase exposure to natural light during lengthening days in summer, or less near-work and more outdoor activities in summer [46]. However, possible variations in seasonal diet were not controlled for. This protective trend of exposure to natural light is based on the assumption that such exposure increases the release of dopamine in the retina, a neurotransmitter known to reduce eye growth in experimental studies [47, 48]. Although these findings are from animal models, they are consistent with the results of study in humans.

### Near-work and myopia

On-screen and reading activities requiring near vision are described as a risk factor for myopia [49]. Here, we detected a marginal effect of screen time, although the contribution of reading time did not seem a significant risk factor. The absence of an effect of reading time could be due to the relatively young age of the children (32% were < 7 years old; Table 2) with high probability of illiteracy. The association between near-work and myopia could also be due to people with myopia engaging in more near-work because taking part in some sports might be difficult when wearing spectacles. A prospective study reported that myopic children may be more at risk of lower levels of physical activity than their non-myopic peers [50]. However, we did not find a significant effect of sport practice on myopia.

### Prevalence of myopia

Variations in the prevalence of myopia by geographical location are well documented [6, 49, 51]. However, the prevalence of myopia in French children has been less studied than in other countries, with limited current data available to understand its evolution in the context of the worldwide increase in myopia incidence. Overall, the proportion of myopic patients in our initial sample reached 32% (38% for girls, 28% for boys). The lower prevalence of myopia in boys is consistent with data from other countries [13, 52]. However, the representativeness of our sample relative to the global paediatric population in France is probably biased because data were collected from hospital consultations, and recruitment included many strabismus patients who were potentially hyperopic in the context of accommodative strabismus.

### Limitations

The size and diversity of the population studied is one of the main limitations. Patients were recruited during medical consultations, which implies some selection bias. Moreover, although the composition of dietary intake varies between age 4 and 18 years, only one food frequency questionnaire was used. In addition, the subjective measurement of refined carbohydrates intake through questionnaire is another limitation. A larger cohort and a food frequency questionnaire that is more age-appropriate will be required to confirm and refine our results.

## Conclusion

This study supports the findings of recent research on risk factors for myopia development and brings new results for the potential effect of refined carbohydrates consumption on this visual disorder. Further prospective studies are needed to confirm these findings and to disentangle the mechanisms by which diet can affect myopia. This study also reinforces the belief that modifiable risk factors for myopia could be targets for future public health actions in France and around the world.

## Supplementary information

**Additional file 1.** Food frequency questionnaire.

**Additional file 2.** Original version of food frequency questionnaire.

## Data Availability

The datasets used and/or analysed during the current study are available from the corresponding author on request.
