# Supplementary material for: Probability of myopia in children with high refined carbohydrates consumption in France
Source: BMC Ophthalmol. 2020 Aug 18;20:337. doi: 10.1186/s12886-020-01602-x (PMC7433090; doi:10.1186/s12886-020-01602-x)
Supplement: Supplementary file 1 — Additional file 1. Food frequency questionnaire. [file 12886_2020_1602_MOESM1_ESM.docx]

**Additional file 1. Food frequency questionnaire**

How often does your child consume the following foods or beverages, regardless of how they are stored (fresh, canned or frozen), when they are eaten (meals or between meals) and where they are consumed (home or out of home) ?

| Foods: | Never or almost never | Less than once a week | 1 time per week | 2-3 times per week | 4-6 times per week | Every day |
| --- | --- | --- | --- | --- | --- | --- |
| Meat, poultry, egg, fish |  |  |  |  |  |  |
| Milk |  |  |  |  |  |  |
| Unsweetened dairy products (cheese, fresh cheese, yogurt, white cheese,…) |  |  |  |  |  |  |
| Sweetened dairy products (cheese, fresh cheese, yogurt, white cheese,…) (already sweet or in which your child adds sugar, honey, jam, compote...) |  |  |  |  |  |  |
| Sweet desserts (dessert cream, mousse, ice cream, compote, fruit in syrup), ... |  |  |  |  |  |  |
| White bread, rusks, crackers, sandwich bread,... |  |  |  |  |  |  |
| Wholemeal bread, buckwheat bread, rye bread, wholemeal rusks, |  |  |  |  |  |  |
| Breakfast cereals |  |  |  |  |  |  |
| Muesli without added sugar |  |  |  |  |  |  |
| White starchy foods (pasta, rice, potato, semolina, flour, ...) |  |  |  |  |  |  |
| Wholemeal starchy foods (wholegrain pasta, rice, semolina, flour, ...) |  |  |  |  |  |  |
| Legumes (lentils, dry beans, chickpeas,...) |  |  |  |  |  |  |
| Vegetables (raw or cooked) |  |  |  |  |  |  |
| Fruits (excluding pressed fruit juices) |  |  |  |  |  |  |
| Ready to eat food |  |  |  |  |  |  |
| Fast food products (hamburgers, pizzas, quiches,…) |  |  |  |  |  |  |
| Fried food (french fries, chips, donuts,...) |  |  |  |  |  |  |
| Aperitif snacks |  |  |  |  |  |  |
| Cookies |  |  |  |  |  |  |
| Nuts (peanuts, walnuts, hazelnuts, almonds,...) |  |  |  |  |  |  |
| Cakes, pastries, viennoiseries |  |  |  |  |  |  |
| Chocolate or cereal bars |  |  |  |  |  |  |
| Sweets |  |  |  |  |  |  |
| Jam, cocoa, honey, sugar |  |  |  |  |  |  |
| Sugar sweetened beverages (cola, ice-tea,...) |  |  |  |  |  |  |
| Fruit juices (pressed or not, with or without added sugar) |  |  |  |  |  |  |
