# Supplementary material for: Probability of myopia in children with high refined carbohydrates consumption in France
Source: BMC Ophthalmol. 2020 Aug 18;20:337. doi: 10.1186/s12886-020-01602-x (PMC7433090; doi:10.1186/s12886-020-01602-x)
Supplement: Supplementary file 2 — Additional file 2. Original version of food frequency questionnaire. [file 12886_2020_1602_MOESM2_ESM.docx]

**Additional file 2.** **Original version of food frequency questionnaire.**

Habituellement, à quelle fréquence votre enfant consomme-t-il les aliments ou boissons suivants, quel que soit leur mode de conservation (frais, en conserve ou surgelé), le moment de consommation (repas ou hors repas) et le lieu (domicile ou hors domicile) ?

| Aliments : | Jamais ou presque | Moins d'1 fois par semaine | 1 fois par semaine | 2 à 3 fois par semaine | 4 à 6 fois par semaine | Tous les jours |
| --- | --- | --- | --- | --- | --- | --- |
| Viande, volaille, œufs, poissons, charcuterie |  |  |  |  |  |  |
| Lait |  |  |  |  |  |  |
| Produits laitiers (fromage, petits suisse, yaourt, fromage blanc,…) non sucrés |  |  |  |  |  |  |
| Produits laitiers (fromage, petits suisse, yaourt, fromage blanc,…) sucrés (déjà sucrés ou dans lesquels votre enfant rajoute sucre, miel, confiture, compote...) |  |  |  |  |  |  |
| Desserts sucrés (crème dessert, mousse, glace, entremet, compote avec sucre ajouté, fruits au sirop), ... |  |  |  |  |  |  |
| Pain blanc, biscottes, pain de mie blanc, tartines craquantes, pains suédois |  |  |  |  |  |  |
| Pain complet, intégral, au sarrasin, au seigle, biscottes complètes |  |  |  |  |  |  |
| Céréales pour le petit-déjeuner |  |  |  |  |  |  |
| Muesli sans sucre ajouté |  |  |  |  |  |  |
| Féculents blancs (pâtes, riz, pomme de terre, semoule, farine, ...) |  |  |  |  |  |  |
| Féculents de type complet (pâtes complètes, riz complet, semoule complète, farine complète,...) |  |  |  |  |  |  |
| Légumes secs (lentilles, haricots secs, pois-chiche,...) |  |  |  |  |  |  |
| Légumes crus ou cuits |  |  |  |  |  |  |
| Fruits frais (hors fruits pressés) |  |  |  |  |  |  |
| Plats cuisinés du commerce (en conserve, surgelés, traiteur,...) |  |  |  |  |  |  |
| Plats de restauration rapide (hamburgers, pizzas, quiches,…) du commerce (surgelés ou frais). |  |  |  |  |  |  |
| Aliments frits de type frites, chips, beignets,... |  |  |  |  |  |  |
| Biscuits salés |  |  |  |  |  |  |
| Biscuits sucrés |  |  |  |  |  |  |
| Graines (cacahuètes, noix, noisettes, amandes,...) |  |  |  |  |  |  |
| Biscuits, gâteaux, pâtisseries, viennoiseries |  |  |  |  |  |  |
| Barres chocolatées, de céréales |  |  |  |  |  |  |
| Bonbons |  |  |  |  |  |  |
| Confiture, cacao, miel, sucre en poudre ou en morceaux |  |  |  |  |  |  |
| Sodas, boissons aromatisées sucrées (ice-tea, oasis,...) |  |  |  |  |  |  |
| Jus de fruits |  |  |  |  |  |  |
